# Growth performance and carcass traits of growing and finishing pigs fed diets with a partial to total replacement of soybean meal with Spirulina powder

**DOI:** 10.1186/s40104-025-01197-7

**Published:** 2025-06-01

**Authors:** Gregorio Don, Diana Giannuzzi, Alessandro Toscano, Stefano Schiavon, Luigi Gallo

**Affiliations:** Department of Agronomy, Food, Natural Resources, Animals and Environment (DAFNAE), University of Padova, Padova, Legnaro (PD) 35020 Italy

**Keywords:** Carcass traits, Growing-finishing pigs, Growth performance, Meat quality attributes, Novel protein sources, Spirulina

## Abstract

**Background:**

The protein sources in pig diets strongly rely on soybean meal, but its production has been associated with soil degradation, deforestation and loss of biodiversity. Microalga Spirulina can be a potentially more sustainable alternative to soybean meal, but comprehensive information about its use in growing pigs is still lacking. This study aimed to evaluate the effects of partial to full replacement of dietary soybean meal with Spirulina on the growth and carcass traits of growing pigs and on the chemical and physical attributes of the meat.

**Methods:**

Eighty-eight pigs, gilts and barrows mixed together, with initial body weight of 52.4 ± 4.2 kg, were allotted into 4 isoenergetic, isoproteic, and isoaminoacidic dietary treatments, which included a conventional control diet based on cereals and soybean meal and one of 3 diets formulated by replacing nearly 33%, 66% or 100% soybean with Spirulina. Each treatment had 2 pens (11 pigs/pen), which were equipped with electronic feeders that were able to record individual feed intake. After 138 d on feed, at 174.9 ± 6.4 kg body weight, the pigs were slaughtered, and the carcass traits and meat quality parameters from loin samples were assessed.

**Results:**

The palatability of feeds was not depressed in pigs fed Spirulina, even when the soybean was completely replaced by the microalga. The incorporation of Spirulina in the diets in place of soybean did not impair the growth rate or feed efficiency, irrespective of the extent of replacement. The carcass traits and yield of commercial cuts were comparable for all Spirulina-included compared with those of the soybean-based groups, and the same was found for the chemical and physical attributes of loin meat.

**Conclusion:**

The results obtained at the herd and slaughter levels revealed that the replacement of soybean meal with Spirulina did not negatively affect the growth or carcass traits of growing pigs or the main attributes of meat. Therefore, this study provides, for the first time, insights into the technical possibility of switching growing pig feeding systems toward more environmentally sustainable diets by including a microalga originating from landless feed production systems, which does not result in soil degradation or loss of biodiversity.

## Background

Livestock production is undoubtedly an important source of benefits, providing food of high nutritional quality and raw material for the agro-food industry, thus playing a significant role in the economies of several countries. However, it is facing arduous environmental sustainability challenges due to the emission of pollutants that contribute to greenhouse, acidification and eutrophication processes, the overuse of water, the loss of biodiversity, and the large use of agricultural land for feed production, which is in direct competition with human food production [1, 2].

The pig sector is a large contributor to global meat yield, accounting for nearly 35% of primary meat production [3], and its contribution to different sources of environmental impact arising from livestock production is therefore substantial [4]. Feedstuff production, on farm but above all off farm, has been identified as the single step of overall pig unit management, most strongly associated with different impact categories [5].

The protein sources used for diet preparation largely rely on soybean meal [6, 7], which is viewed as a particularly damaging crop because its production has been associated with increased soil degradation and deforestation in endangered ecosystems and biodiversity hotspots [8, 9]. Therefore, investigating alternative and/or novel protein sources has become a relevant research topic for improving the sustainability of pig chains. The exploration of alternative protein sources should consider feeds characterized by one or more of the following attributes: local, landless, circular [10]. Among landless novel feeds, microalgae are gaining interest because of their potential competitive advantages.

Microalgae are photosynthetic aquatic microorganisms comprising prokaryotic cyanobacteria and eukaryotic members [11]. As they are produced in water culture or raceway ponds, their cultivation does not require arable land [12], thus contributing to pig production sustainability by reducing land use and degradation and decreasing competition with human food [13]. Microalgae are appreciated for their high protein content, valuable amino acid profile and richness of bioactive peptides, which provide several positive properties [14]. Among these microorganisms, *Arthrospira platensis*, commonly known as Spirulina (SP) has been recognized as particularly interesting as a food and feed, accounting for the largest value of the microalgae market [15]. Spirulina is a cyanobacterium characterized by a protein content ranging between 50% and 70% on dry weight [14]. Moreover, its amino acid profile is comparable to or better than that of soybean meal (SBM) [16], highlighting its potential as an alternative protein source in pig nutrition.

Despite the profusion of interest declared for SP meal in recent years, studies dealing with the use of this microalga in pig nutrition are still scarce. Some studies have used SP as a dietary supplement in minimal amounts in the diets of weaning or growing pigs, with controversial effects on growth performance [17–19]. Only a few studies have included SP as an ingredient in the diets of growing pigs. Martins et al. [20] reported a decrease in the growth rate of postweaning pigs fed for 28 d diets with nearly 50% SBM replaced with SP compared with conventional full-soybean diets. Altmann et al. [21] did not observe effects on most physicochemical and sensory attributes of meat from pigs fed from 20 to 110 kg body weight conventional diets or diets where 50% SBM was replaced by SP meal, but they did not report data on the growth performance of pigs.

Therefore, aside from further economic considerations, the practical feasibility of replacing SBM with SP in the diets of growing pigs and the level of this replacement still need to be adequately addressed. This study aims to evaluate the effects of partial to full replacement of dietary SBM with SP on growth and carcass traits and on the meat quality of growing and finishing pigs.

## Methods

### Animals and facilities

The study involved 88 pigs (37 gilts and 51 barrows) born on the same day in a commercial sow farm and belonging to a genetic line aimed to produce heavy pigs intended for typical dry-cured ham production (Goland C21 × Camborough 43 PIC Italy). They were selected from a batch of 700 pigs’ offspring of 80 sows by minimizing their relationship. Pigs were housed in pens of 5.8 m × 3.8 m with fully slatted floors (2.00 m^2^/pig). Each pen was equipped with a single-space electronic feeder (Compident MLP 2—SMARTCON, Schauer Agrotronic, Prambachkirchen, Austria) programmed to supply each pig with the planned daily amount of feed. For each visit, the station recorded the animal identification, the date and the time of the feeding event, the time spent eating, and the amount of feed consumed. Each station was calibrated weekly by weighing a mass of 1.0 kg, and the standard deviation of the calibration measurements was always within ± 0.005 kg for all stations.

Water was freely available from two nipple drinkers in each pen. The herd was equipped with a ventilation system and dataloggers for recording temperature and relative humidity. Each pen was equipped with two collective straw feeders and two other enrichment tools (beech wood provided with a dispenser fastened to the wall and a hard plastic ball).

Two pigs were removed from the trial for injuries, and the corresponding data were excluded, resulting in a final dataset of 86 pigs.

### Experimental design, feeds and feeding

Pigs were moved to the experimental pig unit of the DAFNAE Department of the University of Padova at the age of 82 d, and a body weight (BW) of 41.3 ± 3.4 kg. They were individually identified with an ear tag upon arrival and homogeneously allotted to 8 pens (11 pigs/pen, 2 pens per treatment) on the basis of their initial BW, with gilts and barrows mixed together (4 to 6 gilts and 5 to 7 barrows per pen). After 15 d of acclimation to pens and feeding stations, when the BW of pigs averaged 52.4 ± 4.2 kg, pigs were allocated to four dietary treatments (2 pens per treatment): a control diet (SP00), which was based on cereals and SBM, and three experimental diets formulated by replacing 33% (SP33), 66% (SP66) or 100% (SP100) SBM with a nucleus based on spray-dried *Arthrospira platensis* powder (Aim Grow Biotech Co., Ltd., Port Coquitlam, BC, Canada).

The SP00 feeds used in the early (from 50 to 90 kg BW) and late growing (from 90 to 140 kg BW) and finishing periods (from 140 to 170 kg BW) were conventional diets representative of those commonly used for Italian heavy pigs aimed at typical ham production. The experimental diets were formulated to be tested against SP00 by replacing a progressively greater amount of SBM with a SP nucleus. This nucleus (Table 1) was specifically formulated to mirror the soybean meal for its major nutrient contents because the SP used in this study had a greater crude protein content than SBM accounting 66% and 45% as-fed, respectively. In this way, we were able to obtain comparable contents of energy, protein and main essential amino acids in all the diets from the same feeding period (Table 2). The feeds used within a feeding period were produced using the same batches of feed ingredients, and all the feeds were manufactured by the same feed factory (Neviani Mangimi SRL, Montecchio Emilia, Reggio Emilia, Italy). The ingredient compositions of the feeds are reported in Table 1, whereas the chemical compositions and nutrient contents are given in Table 2.

**Table 1:** Ingredient composition of experimental feeds, g/kg as-fed

Ingredients	Early growing feeds (50–90 kg BW)				Late growing feeds (90–140 kg BW)				Finishing feeds (140–170 kg BW)			
	SP00	SP33	SP66	SP100	SP00	SP33	SP66	SP100	SP00	SP33	SP66	SP100
Corn grain	527.6	526.8	526.1	525.4	527.0	526.4	525.8	525.2	527.1	526.6	526.1	525.6
Barley grain	159.6	159.3	159.1	158.9	189.3	189.1	188.9	188.6	224.6	224.4	224.2	224.0
Wheat middlings	60.3	60.2	60.1	60.1	50.2	50.1	50.1	50.0	50.4	50.3	50.3	50.2
Wheat bran	29.6	29.5	29.5	29.5	29.6	29.5	29.5	29.5	29.6	29.5	29.5	29.5
Lard	10.3	10.3	10.3	10.3	10.3	10.3	10.3	10.3	10.3	10.3	10.3	10.3
SP Nucleus^a^	0.0	62.1	124.1	187.9	0.0	51.9	103.7	157.3	0.0	40.7	81.4	121.9
Soybean meal	184.5	123.5	62.7	0.0	154.4	103.5	52.7	0.0	119.6	79.6	39.8	0.0
Calcium carbonate	16.0	15.9	15.9	15.9	16.0	15.9	15.9	15.9	16.0	15.9	15.9	15.9
Silica^b^	-	-	-	-	11.1	11.1	11.1	11.0	11.1	11.1	11.1	11.1
Sodium chloride	4.4	4.4	4.4	4.4	4.4	4.4	4.4	4.4	4.4	4.4	4.4	4.4
Dicalcium phosphate	4.3	4.3	4.3	4.3	4.3	4.3	4.3	4.3	4.3	4.3	4.3	4.3
Vitamin mineral premix^c^	1.7	1.7	1.7	1.7	1.7	1.7	1.7	1.7	1.7	1.7	1.7	1.7
L-Lysine HCl^d^	0.7	0.7	0.7	0.6	0.7	0.7	0.7	0.6	0.0	0.0	0.0	0.0
Optiphos (phytase)^e^	1.1	1.1	1.1	1.1	1.1	1.1	1.1	1.1	1.1	1.1	1.1	1.1

*BW* Body weight

*SP00* control diets without Spirulina inclusion

*SP33, SP66 and SP100* experimental diets with 33%, 66% and 100% replacement of soybean meal with Spirulina nucleus

*SP Nucleus* Spirulina powder nucleus

^a^Ingredient composition: *Arthrospira platensis* 640 g/kg; Sugar beet pulp 308 g/kg; Soft wheat 40 g/kg; L-Lysine monohydrochloride 6 g/kg; L-triptophan 6 g/kg. Chemical composition (g/kg): Dry matter 913; Crude protein (N × 6.25) 467; Lysine 28; Methionine + cysteine 13.2; Threonine 21.5; Triptophan 6.9; Starch 25; Ether extract 47; Ash 64

^b^Silica (Silica granular 10.SiO2.H2O, Impextraco, Heist-op-den-Berg, Belgium) included to increase the acid-insoluble ash content as a marker for a further study aimed to assess the digestibility of diets based on different level of SP

^c^Providing per kilogram of feed: vitamin A, 8,000 IU; vitamin D_3_, 1,200 IU; vitamin E, 8 mg; Vitamin B_7_, 0.08 mg; vitamin B_12_, 0.012 mg; niacin, 16.0 mg; biotin, 8 mg; iron, 170 mg; zinc, 117 mg; copper, 14 mg; cobalt, 0.11 mg; iodine, 0.06 mg; manganese, 65 mg; magnesium, 0.14 mg; selenium 10 mg

^d^L-Lysine monohydrochloride, 98.5% pure, 78% L-Lysine (Methodo Chemicals, 42017 Novellara, RE, Italy)

^e^OptiPhos® (Phytase, Huvepharma)

**Table 2:** Nutrient composition (g/kg as-fed, unless otherwise indicated) and color parameters of experimental feeds

Item	Early growing feeds (50–90 kg BW)				Late growing feeds (90–140 kg BW)				Finishing feeds (140–170 kg BW)			
	**SP**00	**SP**33	**SP**66	**SP**100	**SP**00	**SP**33	**SP**66	**SP**100	**SP**00	**SP**33	**SP**66	**SP**100
Nutrient composition^a^												
Dry matter	906	910	917	921	922	920	922	924	899	899	901	902
Crude protein	155	156	157	157	149	145	146	147	130	131	131	132
Ether extract	36	39	44	47	40	41	42	46	38	39	40	39
NDF	123	116	118	120	130	125	118	116	142	137	139	138
Starch	503	477	461	466	399	386	392	398	404	404	406	404
Ash	49	49	49	49	54	52	54	53	43	50	45	46
Lysine	8.3	8.5	8.4	8.4	8.0	8.2	8.1	8.1	6.6	6.5	6.7	6.9
Methionine	2.3	2.7	3.2	3.2	2.2	2.7	3.1	3.3	2.2	2.5	2.4	2.8
Threonine	5.0	5.4	5.6	5.7	4.8	5.2	5.6	5.7	4.3	4.5	4.6	5.0
Tryptophan	1.4	1.6	1.7	1.9	1.3	1.5	1.6	1.9	1.2	1.1	1.3	1.4
Tyrosine	2.6	2.7	2.8	2.9	2.4	2.6	2.8	2.9	2.3	2.4	2.4	2.6
Ca	10.0	9.6	8.7	9.3	9.3	8.7	9.4	10.8	8.6	10.4	9.9	9.1
P	4.0	4.1	4.0	4.1	4.6	4.7	4.7	4.6	4.3	4.5	4.6	4.6
Na	0.8	1.4	1.2	1.1	2.2	1.9	2.3	2.0	1.8	1.9	1.9	2.0
Computed energy content^b^, MJ/kg												
Metabolisable energy	13.4	13.4	13.4	13.5	13.2	13.3	13.3	13.4	13.2	13.3	13.3	13.3
Net energy	9.9	10.0	10.0	10.0	9.9	9.9	10.0	10.0	10.0	10.0	10.0	10.0
Fatty acid composition, % total fatty acids												
Saturated fatty acids	24.40	24.89	26.21	26.59	23.98	24.95	25.72	26.63	23.62	24.93	25.07	25.21
Monounsaturated fatty acids	32.26	31.52	30.45	30.63	32.70	32.57	32.49	32.46	32.43	32.72	31.94	31.41
Polyunsaturated fatty acids	43.35	43.59	43.34	42.78	43.33	42.48	41.79	40.92	43.95	42.35	42.99	43.38
Color parameters of feeds^c^												
CIE L*	-	-	-	-	78.43	63.58	56.79	51.85	-	-	-	-
CIE a*	-	-	-	-	3.11	-0.90	-0.99	-0.70	-	-	-	-
CIE b*	-	-	-	-	19.80	17.22	16.63	16.35	-	-	-	-
Chroma (c)	-	-	-	-	20.05	17.24	16.66	16.37	-	-	-	-
Hue (h)	-	-	-	-	81.08	92.99	93.41	92.44	-	-	-	-

*BW* Body weight

*SP00* Control diets without Spirulina inclusion

*SP33, SP66 and SP100* Experimental diets with 33%, 66% and 100% replacement of soybean meal with Spirulina nucleus

*CIE* Commission Internationale de l’Eclairage

^a^Analytical results are the average of 2 independent replications

^b^Computation based on the NRC [22] tabular values of each feed ingredient, assuming for Spirulina powder the same energy content of soybean meal

^c^Determination performed with Minolta colorimeter CM-600d (Konica Minolta Corp., Japan)

Pigs were fed pelleted feeds on the basis of a mildly restricted feeding scale adjusted at 2-week intervals, and the amount of feed provided ranged from 1.90 to 3.20 kg/d from the first to the last week on feed. The change from early- to late-growing diets occurred at 92 ± 5.5 kg BW after 42 d on feed, and the change to finishing diets occurred at 142 ± 5.7 kg BW after 98 d on feed.

### Feeds analysis

Ten samples of each diet were collected online during the manufacturing of the feeds. The samples were separately pooled, mixed and sampled again to obtain 1 kg of sample for each feed. This method has been used to collect independent subsamples that have been analyzed in 2 replications to determine the content of dry matter (DM: # 934.01; [23]), crude protein (CP: # 976.05; [23]), ether extract (EE: cold extraction in a separating funnel using chloroform (2:1); [24]), ash (# 942.05, [23]), and neutral detergent fiber with amylase [25]. The starch content was determined after hydrolysis to glucose via liquid chromatography [26]. The amino acid contents of the samples were determined according to the Council of Europe [27].

The color of the feeds from the late growing period was measured using a Minolta colorimeter CR-600d (Konica Minolta Corp., Japan) equipped with a D65 illuminant and at a 10° angle of observation, in accordance with the CIE L*, a*, b* color system [28]. The colorimeter was previously calibrated, and the average of 3 random readings was used to measure L* (lightness, from 0, dark, to 100, white), a* (redness) and b* (yellowness). Additionally, chroma (C*) and hue angle (h°), defined as color intensity and saturation, respectively, were obtained by using the following equations: C = (a^2^ + b^2^)^1/2^ and h° = arctg b*/a* [29].

### On-farm traits

Individual BW was recorded every 2 weeks using an electronic scale (Tru-Test S3 Weigh System with MP600 Loadbars, Datamars Agri UK Ltd., Selkirk, UK). The backfat depth (BF) was measured in conjunction with BW recording above the last rib at approximately 5.5–8.0 cm from the midline, according to body size, and considering both the first and second layer using an A-mode ultrasonic device (Renco Lean-Meater Series 12, Renco Corporation, Minneapolis, MN, USA) [30]. Individual feed intake was collected daily from the electronic feeders. The average daily gain (ADG) was estimated through individual linear regressions of BW on days on feed (average *R*^2^ of the regression: 0.996 ± 0.003, minimum *R*^2^: 0.982). The average daily feed intake was computed by dividing the individual cumulative feed intake during the trial by the days on feed from the beginning to the end of the trial. The feed efficiency was expressed as the gain-to-feed ratio (G:F).

Fecal grab samples were collected from each pig during the late growing period. Samples were dried for 48 h at 60 °C, ground through a 2-mm screen, and assessed for the colour using the same Minolta colorimeter and the same procedure described previously for the determination of feed color.

### Carcass traits and meat samples

After 138 d on feed, when the average BW was 174.9 ± 6.4 kg, all the pigs were slaughtered after a 17-h fast, and the carcasses were processed according to conventional practices used for preparing Italian heavy pigs and cuts [31]. Pigs were electrically stunned and exsanguinated, and the carcasses were scalded, dehaired, eviscerated and split down the midline of the spine, maintaining individual identification throughout the slaughtering chain. The hot weight of the carcasses was recorded online after the removal of the head. Backfat thickness was measured online on the left side of the carcasses at 7 cm from the midline using a Hennessy grading probe (Hennessy Grading Systems, Auckland, New Zealand).

The warm carcasses were dissected, and the weights of the loins with ribs, lards and green hams were recorded. After a 24-h chilling period (0 to 2 °C), the green hams were dressed to obtain the typical round ham shape and weighed again.

A section of *longissimus lumborum* (LL), including the 3^rd ^and 4^th^ thoracic vertebrae, was collected from the left loin of each carcass and transferred refrigerated (4 °C) to the meat laboratory in vacuum-packaged bags.

### Physical and chemical analyses of meat

Samples of LL were deboned after 24 h of chilling at 4 °C and divided into two aliquots, one analyzed for physical parameters and the other for chemical parameters.

Physical analyses on LL concerned:

- *pH*, measured in triplicate at 24 h postmortem using a bench pH meter (Sension pH meter, Hach Company, Colorado, USA) equipped with a 50 53 T penetration probe with a built-in thermometer for pH correction;
- *meat color* was measured using a Minolta Chromameter set (Minolta CM-600d, Konica Minolta Sensing Americas, Inc., New Jersey, USA) equipped with a D65 (noon daylight, 6500 K) illuminant d/10° (diffuse illumination/10° angle of observation) geometry and an 8 mm diameter circular aperture, according to the procedures described by AMSA [29]. The instrument was calibrated by scanning black and white standardized tiles. The average of 3 readings was used to measure L* (lightness), a* (redness) and b* (yellowness), whereas Chroma (C*) and the hue angle (h°) were computed using the equations reported above;
- *cooking losses*, determined on a 2.5 cm thick subsample after treatment in a water bath cooking for 50 min until the core temperature reached 70 °C. The cooked samples were subsequently cooled to room temperature, blotted dry, and reweighed. The cooking loss percentage was calculated by dividing the weight difference before and after cooking by the precooked weight;
- *Allo-Kramer shear force resistance, *measured using an LS5 electronic dynamometer (Lloyd Instruments, AMETEK Inc., Berwyn, Pennsylvania, USA) equipped with a load cell and an Allo-Kramer shear cell (width 70 mm, thickness 2 mm), with 10 flat-bottomed blades. Pieces of size 7 cm × 3 cm × 1 cm obtained from the sample used for the determination of cooking losses were cut with a sliding speed of the cutting apparatus of 500 mm/min [32]. The cutting work, calculated as the work from the beginning of the test to the maximum peak load (defined as the maximum force required to cut the sample), was determined using the Nexygen Plus 3 software (Lloyd Instruments, AMETEK Inc., Berwyn, Pennsylvania, USA).

For chemical analysis, the second aliquot of LL was ground, mixed, and homogenized for 10 s at 4,500 × *g* using a Grindomix GM200 (Retsch, Haan, Dusseldorf, Germany) and analyzed with 2 independent replicates for moisture content (#950.46; [23]), crude protein (CP) according to the Kjeldahl method [23], fat content after extraction with petrol ether (# 991.36; [23]) and ash through mineralization (# 920.153; [23]).

For the determination of the fatty acid (FA) profile, the fat extracted was methylated according to Christie [33]. Methylated fatty acids were analyzed via GC-FID with an Agilent 7820A gas chromatograph (Agilent Technologies, Santa Clara, CA, USA). One microliter was injected with a split ratio of 65:1. A Supelco OMEGAWAX-TM 250 (Sigma‒Aldrich, St. Louis, MO, USA; 30 m × 0.25 mm internal diameter, 0.25 μm film thickness) was used with hydrogen as the carrier at 1.4 mL/min to separate methylated fatty acids. The oven temperature was set at 50 °C, held for 2 min, increased to 220 °C at a rate of 4 °C/min, and then held for 23 min. Both the injector and the detector temperatures were set at 250 °C. The individual FAs were identified by comparing the retention times of the standard FA methyl ester mixtures (Supelco 37–component FAME Mix, 47885–U). The FA composition has been reported as total, saturated, monounsaturated and polyunsaturated, expressed as grams per 100 g of total FA.

### Statistical analysis

The data were analyzed using the MIXED procedure of SAS (SAS Inst. Inc., Cary, NC, USA) according to the following linear model: 

$$y_{ijkl} = \mu + \text{Diet}_{i} + \text{Sex}_{j} + (\text{Diet} \times \text{Sex})_{ij} + \text{Pen}(\text{Diet})_{k:i} + \varepsilon_{ijkl}$$

where *y*_*ijkl*_ is the observed trait; *μ* is the overall intercept of the model; Diet_*i*_ is the fixed effect of the *i*^th^ treatment (*i* = 1…, 4); Sex_*j*_ is the fixed effect of the *j*^th^ sex (*j*: 1 = gilts, 2 = barrows); *(*Diet × Sex)_*ij*_ is the interaction effect between dietary treatment and sex; Pen(Diet)_*k:i*_ is the random effect of the *k*^th^ pen within the diet; and *ε*_*ijkl*_ is the random residual error. Pen and the residual were assumed to be independently and normally distributed with a mean of zero and variances of *σ*^*2*^_*k*_ and *σ*^*2*^_*e*_, respectively. The effect of diet was tested on the error line of the pen within diet, whereas sex and the diet × sex interaction were tested on the residual variance. Differences between the least squares means (LSmeans) of the different diets were adjusted using the Bonferroni correction method and considered significant at a *P* value ≤ 0.05. When the *P* value of the diet effect was ≤ 0.05, polynomial contrasts were estimated between the least square means of the diets to examine the response curve of each trait (linear, quadratic and cubic components) due to the progressive replacement of soybean meal with SP.

## Results

The interaction effect of diet × sex was not significant for any of the growth or carcass traits or for the physical or chemical attributes of the meat. Therefore, only the results concerning the main effects of diet and sex will be considered hereinafter.

The least square means of growth performance traits of pigs fed different levels of SP in the diets are reported in Table 3. The average BW of pigs at the beginning and end of the trial was approximately 52 and 174 kg, respectively, with an average BW gain close to 0.9 kg/d. The average feed intake was around 2,600 g/d, and the pigs gained around 340 g BW/kg of feed consumed.

**Table 3:** Least squares means of growth traits and fecal colour parameters of heavy pigs fed different levels of Spirulina

Item	Sex (S)			Diet (D)					*P*_S x D_	RMSE
	Gilts	Barrows	*P*	SP00	SP33	SP66	SP100	*P*		
Pigs, n	36	49		22	21	21	21			
Body weight, kg										
Initial	52.1	52.8	0.760	51.1	53.0	53.0	52.7	0.759	0.184	3.98
Final	174.6	172.5	0.478	174.8	171.6	173.0	174.7	0.471	0.790	6.48
Daily gain, g/d	904	886	0.029	913	881	881	904	0.409	0.877	36.0
Ultrasound backfat thickness, mm										
Initial	7.8	7.9	0.460	7.6	8.1	7.8	8.0	0.871	0.070	0.90
Final	16.8	16.8	0.918	16.7	16.3	17.2	17.0	0.896	0.914	1.76
Feed intake, g/d	2,600	2,637	< 0.001	2,610	2,598	2,642	2,625	0.657	0.418	47.5
Gain to feed, g/g	0.348^b^	0.330^a^	< 0.001	0.350	0.339	0.334	0.344	0.347	0.457	0.013
Fecal colour parameters										
CIE L*	47.09	46.94	0.576	57.46^d^	47.88^c^	43.02^b^	39.71^a^	< 0.001	0.126	1.149
CIE a*	3.30	3.24	0.497	4.74^b^	2.77^a^	2.67^a^	2.89^a^	< 0.001	0.624	0.395
CIE b*	16.96	16.78	0.232	16.88^ab^	17.55^b^	16.83^ab^	16.23^a^	0.035	0.990	0.658
Chroma (c)	17.31	17.11	0.228	17.53^ab^	17.77^b^	17.04^ab^	16.49^a^	0.026	0.996	0.695
Hue (h)	79.02	79.12	0.679	74.33^a^	81.06 ^b^	80.99^b^	79.91^b^	0.001	0.466	1.074

*SP00* control diets without Spirulina inclusion

*SP33, SP66 and SP100* experimental diets with 33%, 66% and 100% replacement of soybean meal with Spirulina nucleus

*CIE* Commission Internationale de l’Eclairage

^a–d^Means within a row without common lower case letters differ (*P* < 0.05)

Diets did not affect any of the growth traits considered, and pigs fed diets with different contents of SP presented feed intake, daily gain and feed efficiency comparable with those of the SP00 group, regardless of the level of inclusion of SP in the diet. As a result, the least square means of final BW were very similar among pigs in the different dietary treatments, and the differences among the groups were nominal and did not exceed 3 kg on average. Additionally, the ultrasound backfat thickness was fully comparable among the different experimental groups both at the beginning and at the end of the trial, and consequently, the gain in backfat during the study was similar among groups.

Conversely, the color of the feces was significantly influenced by the diet. Specifically, the progressive replacement of SBM with SP resulted in darker feces (*P* < 0.05) as a consequence of the linear progressive decrease in the CIE L* parameter, mirroring what was observed in the color parameters of the feeds (Table 2). Moreover, compared with SP00 pigs, animals fed SP diets produced significantly greener and more intensively colored feces because of their lower CIE a* and greater h parameters, respectively, without significant differences in the level of SP included. Conversely, yellowness and chroma parameters did not provide a systematic differentiation between SBM- and SP-fed pigs.

With respect to sex effects, gilts presented a slightly but significantly (*P* < 0.05) greater growth rate (+ 2%) and lower feed intake (− 1.4%) than barrows did. As a consequence, gilts exhibited also a greater gain-to-feed ratio than barrows (+ 5%, *P* < 0.05), whereas differences in final BW between pigs of different sex were only nominal.

The least square means of the carcass traits of pigs fed different levels of SP in the diets are reported in Table 4. The average carcass weight was approximately 139 kg, and the average carcass weight of loins with ribs and of left trimmed ham were approximately 26 and 13.6 kg, respectively. Loins and left trimmed hams accounted for nearly 19% and 9.8% of the carcass weight, respectively. Again, feeding diets characterized by different levels of SBM replacement with SP affected neither the carcass nor the main commercial cuts. As a consequence, control pigs and animals fed diets containing different amounts of SP had very similar carcass compositions in terms of loins, lards and hams, without any differentiation among different levels of SP inclusion.

**Table 4:** Least squares means of carcass traits of heavy pigs fed different levels of Spirulina

Item	Sex (S)			Diet (D)					*P*_S x D_	RMSE
	Gilts	Barrows	*P*	SP00	SP33	SP66	SP100	*P*		
Pigs, n	36	49		22	21	21	21			
Carcass weight (CW), kg	140.1	138.2	0.137	140.6	137.6	138.6	139.8	0.434	0.935	5.66
Hennessy probe backfat thickness, mm	24.9	26.2	0.049	24.9	26.6	26.3	24.5	0.654	0.554	3.13
Weight of, kg										
Loins	26.65	25.52	0.004	26.64	25.56	26.05	26.07	0.379	0.913	1.748
Lards	8.08	8.68	0.009	8.36	8.38	8.46	8.33	0.994	0.689	0.998
Left trimmed ham, kg	13.78	13.58	0.207	13.80	13.55	13.60	13.78	0.619	0.998	0.705
Incidence on CW of, %										
Loins	19.01	18.44	0.002	18.94	18.57	18.77	18.62	0.731	0.525	0.787
Lards	5.77	6.29	0.001	5.95	6.11	6.10	5.97	0.938	0.687	0.684
Left trimmed ham	9.84	9.82	0.788	9.81	9.85	9.81	9.85	0.960	0.930	0.329

*SP00* control diets without Spirulina inclusion

*SP33, SP66 and SP100* experimental diets with 33%, 66% and 100% replacement of soybean meal with Spirulina nucleus

Conversely, carcasses from barrows were significantly fatter than those from gilts. Indeed, barrows had 5% thicker backfat and 7% heavier lards and, in turn, 4% lighter loins than gilts. Therefore, the effect of loins on carcass weight was nearly 3% lower and that of lards was nearly 9% greater in barrows than in gilts. In contrast, gilts and barrows provided hams of similar weight.

The least square means of the physical and chemical attributes of the *longissimus lumborum* samples of pigs fed different levels of SP in the diets are reported in Table 5. In general, replacing SBM with SP, both partially and in total, did not affect loin attributes, such as pH, Minolta color parameters, the Allo‒Kramer shear force, or meat chemical composition traits. Significant differences in loin cooking losses were observed only between two different levels of SP inclusion in the diet, while meat from SP00 showed intermediate cooking losses and was not significantly different from any of the other experimental groups.

**Table 5:** Least squares means of loin physical and chemical attributes of heavy pigs fed different levels of Spirulina

Item	Sex (S)			Diet (D)					*P*_S x D_	RMSE
	Gilts	Barrows	*P*	SP00	SP33	SP66	SP100	*P*		
Pigs, n	36	49		22	21	21	21			
Loin pH	5.60	5.57	0.365	5.62	5.56	5.59	5.56	0.597	0.424	0.162
Loin color										
CIE L*	44.95	46.79	0.010	46.00	45.44	48.03	44.01	0.198	0.803	3.300
CIE a*	3.75	3.92	0.679	3.49	4.32	3.62	3.91	0.756	0.486	1.835
CIE b*	11.89	12.52	0.060	11.99	12.40	12.58	11.84	0.629	0.196	1.503
Chroma (c)	12.58	13.20	0.153	12.58	13.23	13.19	12.56	0.734	0.256	1.929
hue (h)	73.29	73.34	0.969	74.49	71.59	74.93	72.22	0.641	0.554	6.038
Loin cooking loss, %	28.38	28.04	0.453	27.85^ab^	28.85^ab^	26.12^a^	30.04^b^	0.015	0.101	2.044
Allo-Kramer loin shear peak load, kg/g	8.76	9.35	0.184	9.81	8.59	9.00	8.81	0.594	0.424	1.990
Loin chemical composition, g/100 g										
Humidity	72.91^b^	72.23^a^	0.001	72.48	72.26	72.68	72.85	0.340	0.09	0.924
Crude protein	22.57	22.30	0.145	22.62	22.63	22.38	22.11	0.293	0.515	0.816
Lipids	2.97^a^	3.84^b^	< 0.001	3.22	3.47	3.34	3.57	0.850	0.247	0.952
Ashes	1.15	1.13	0.071	1.14	1.13	1.15	1.13	0.471	0.989	0.048
Loin fatty acid (FA) composition, g/100 g FA										
Saturated FA	36.86	37.24	0.672	37.34	36.84	37.85	36.16	0.737	0.769	2.186
Monounsaturated FA	52.72	52.88	0.645	52.80	53.14	52.52	52.74	0.835	0.138	1.646
Polyunsaturated FA	10.44	9.89	0.139	9.86	10.03	9.63	11.13	0.440	0.346	1.682

*SP00* control diets without Spirulina inclusion

*SP33, SP66 and SP100* experimental diets with 33%, 66% and 100% replacement of soybean meal with Spirulina nucleus

*CIE* Commission Internationale de l’Eclairage

*FA* Fatty acid

^a,b^Means within a row without common lower case letters differ (*P* < 0.05)

In terms of sex effects, sample loins from barrows evidenced a greater (*P* < 0.05) lightness than gilts did and had nearly 30% greater intramuscular fat content, showing also a lower meat humidity content.

## Discussion

### General considerations and sex effects

The recognition of novel and sustainable protein sources to replace soybean meal in the diets of growing pigs is considered strategic for the pig sector in recent years. The microalga *Arthrospira* spp. has attracted increasing interest for several appealing nutritional attributes, but considerations about its potential utilization in commercial feed preparation do require a comprehensive evaluation of animal responses to different levels of microalga inclusion in the diet. To the best of our knowledge, our study is the first to use SP as a feed ingredient to replace partially up to completely SBM in the diets of pigs from the early growing to the finishing period until heavy BW.

The growth performance and feed efficiency results obtained in this study were consistent with the results of others for pigs of this class of body weight at slaughter [34]. The effect of sex was included in this study mainly for revealing potential differences in the response of gilts and barrows to different levels of SP in the diets. However, the interaction effect between sex and dietary treatment was not significant for any of the production or quality traits considered. Therefore, the response of pigs to the progressive replacement of SBM with SP was the same irrespective of sex. The differences observed between gilts and barrows in this study generally agreed with findings reported by other studies. Indeed, Latorre et al. [35] and Malgwi et al. [34] reported that gilts had better feed efficiency than barrows because of a better growth rate, lower daily feed intake or both, as in our study. Moreover, both Latorre et al. [36] and Schiavon et al. [37] reported that barrows provided fatter carcasses and greater yields of fat cuts than gilts, which is consistent with our findings. Last, lower intramuscular fat in *longissimus lumborum* from gilts than in that from barrows, as observed in our study, has been reported, among others, by Babicz et al. [38] in a study aimed to determine the basic chemical composition and mineral content in the sirloin and offal of fattener pigs.

### Progressive replacement of soybean meal with Spirulina in the diets of pigs

Overall, the results from the present research revealed that the inclusion of SP in the diets of growing and finishing pigs in place of SBM, until its full replacement, did not impact the growth traits or feed efficiency of the animals. Comparisons with the results of other trials are difficult, as the literature concerning the use of SP in the nutrition of pigs is still limited. Some studies addressing the use of SP in pig diets have included this microalga as a supplement in weaning piglets, generally with contrasting outcomes. Grinstead et al. [39] supplemented 28- or 42-d diets for weanling piglets with 1 to 20 g/kg SP and reported inconsistent and minimal improvements in growth performance. Similarly, Furbeyre et al. [17] did not report effects on the growth rate and feed efficiency of a diet supplemented with 1% SP for 28 d on weaned piglets. On the other hand, Nedeva et al. [40] reported that supplementation with 0.15% to 0.2% SP in the diet improved the growth rate and feed conversion of piglets from approximately 12 to 30 kg BW, and Furbeyre et al. [18] reported a better growth rate during the suckling period and greater weaning weight in piglets administered 385 mg SP/kg BW for 28 d.

Some studies have investigated the use of SP as a dietary supplement also in the feeding of growing pigs, with generally positive effects on growth parameters. Indeed, Simkus et al. [41] observed a greater growth rate and a better feed conversion index in growing pigs fed 2 g/d SP from 30 to 96 kg BW compared to control groups. Similarly, better daily gain and feed efficiency have been reported by Liu et al. [19] for growing pigs fed diets including 0.05% to 0.10% SP for 6 weeks of trial, in the interval 25 to 55 kg BW.

The literature concerning the use of SP as an ingredient in the diets of pigs is even more limited. Martins et al. [20] fed post-weaned piglets from 12 to 28 kg BW diets which included 10% SP in partial replacement (approximately 60%) of SBM supplemented or not supplemented with carbohydrate-degrading enzymes, to produce a typical local specialty named spit-roasted piglets. They reported that the use of SP significantly decreased the daily gain and carcass weight and worsened the feed efficiency compared with those of the control group irrespective of the inclusion of the enzymes and related these negative results to the increase in digesta viscosity and to a lower protein digestibility of SP diets as a consequence of the resistance of microalgal proteins to the action of endogenous peptidases.

Notably, in this trial we observed that SP inclusion had no significant influence on the digestibility of dry matter and lipids, whereas we observed a slight linear decrease in crude protein digestibility with increasing replacement of SBM with SP, with a maximum reduction of 3.7% in the SP100 group. These results agree with data of Viton and Garcia [42], who reported in a digestibility trial that a 100% replacement of SBM with SP in the diets of barrows of 30 kg BW caused only a slight (2% to 3%) decrease in the digestibility coefficients of organic matter and crude protein without affecting nitrogen retention.

Therefore, we may hypothesize that the possible detrimental effects on nutrient digestibility resulting from the inclusion of a high dosage of SP in the diets of growing pigs could also depend on the weight and degree of maturity of the animals, which may consequently affect the performance response to such inclusion. In this regard, and in agreement with our results, Coelho et al. [43] reported that replacing nearly 40% of SBM with *Chlorella vulgaris*, a freshwater green microalga that has been reported to decrease the total tract apparent digestibility of nutrients in post-weaning piglets [44], did not affect the daily gain and feed efficiency of barrows in the interval of 59 to 101 kg BW.

It is also worth considering that apparently the acceptance of the diets was not modified by the replacement of SBM with SP, as the feed intake was very similar among the experimental groups also for the highest level of SP in the diet. This finding is consistent with the results of Martins et al. [20], who did not observe significant differences in feed intake in piglets fed from 12 to 30 kg diets in which SP replaced nearly 60% of the SBM. Similarly, Coelho et al. [43] reported comparable feed intake for growing pigs fed from 59 to 101 kg control diets or diets with a nearly 40% replacement of SBM with *Chlorella vulgaris*. On the other hand, Velten et al. [45] reported lower acceptance in growing chickens of diets in which 50% SBM was replaced with SP, with a consequent significant decrease in average feed intake.

Also, carcass traits were not influenced by different levels of inclusion of SP in the diets of growing and finishing pigs in place of SBM. Indeed, the effects of backfat thickness and the incidence of main commercial cuts on carcass weight were comparable among the different dietary treatments and did not evidence any consistent trend at increasing the amount of SP in the diets.

When used as a supplement in the diets of growing pigs, Simkus et al. [41] reported that the inclusion of 2 g/d SP did not affect backfat thickness and ham weight. Conversely, Yordanova et al. [46] found that supplementing diets of growing pigs with 2 g/d SP in the BW interval of 35–105 kg modified the carcass composition by increasing the percentage of meat with bones and decreasing the amount of fat in the carcass.

In the only study found in the literature concerning the effects of SP as a feedstuff in the diets of growing pigs, Altmann et al. [21], similar to our results, reported comparable lean meat percentages in the carcasses of pigs fed conventional SBM-based diets and pigs fed diets where 50% to 100% of SBM was replaced by SP. Also, the use of other microalgae as feed ingredients for growing pigs provided results comparable with those observed in our study. Indeed, Coelho et al. [43] observed similar backfat thickness and comparable loin weight in pigs fed diets in which nearly 40% of the SBM was replaced by *Chlorella vulgaris*. On the other hand, the inclusion of SP as a feed ingredient in the diets of broiler chickens had inconsistent effects on carcass composition. Indeed, Spinola et al. [47] recently reported that low levels of SP supplementation, on the order of 0.5%–1% of total feed, were generally associated with improved carcass traits, whereas the response of carcass traits to higher daily intake of SP was less consistent and deserves further research. However, both Altmann et al. [48] and Fernandes et al. [49] reported comparable breast muscle yields in broilers fed conventional SBM diets or diets in which 40% to 50% of the SBM was replaced by SP.

The results of our study revealed that different levels of replacement of SBM with SP did not affect the physical or chemical attributes of loin meat such as color, cooking loss, shear force, and main nutrient contents, compared with those of SBM-based pigs. The use of SP as a feed supplement in growing pigs provided results comparable with our findings concerning meat quality attributes. Specifically, Simkus et al. [41] and Yordanova et al. [46] reported no effects of the supplementation of 2 g/d of SP to the diets of growing pigs on pH, Hunter Lab loin color, cooking loss and meat tenderness, whereas Simkus et al. [41], unlike our study, reported lower intramuscular fat content in SP-supplemented pigs. Also, the few studies that included SP at greater levels by replacing SBM in the formulation of the diets of growing pigs generally did not find an impairment in most meat quality parameters. Martins et al. [20], in piglets aimed to spit roast production, and Altmann et al. [21], in barrows raised to 110 kg BW, reported similar pH values, meat colors, cooking losses, shear forces and main nutrient contents in meat from conventional and SP included diets. Furthermore, the addition of 5% *Chlorella vulgaris* to the diets did not alter the physical or chemical attributes of the meat of growing pigs [43]. Interestingly, no study has reported alterations in the color of meat obtained from growing pigs fed with SP at high dosages, as in our study, whereas when SP was fed as an ingredient to chicken broilers, several studies have described meat color modifications. Zampiga et al. [50], in a trial with broiler chickens fed diets with different levels of replacement of SBM with SP (approximately 30%–60% during growth and 45%–90% during finishing), reported that the most relevant effect of such replacement concerned an increase in yellow pigmentation in the meat, which was also visually perceivable. Similarly, Fernandes et al. [49], using diets incorporating 15% SP as a feed ingredient, reported that the raw breast meat of SP-fed broiler chickens presented a significant increase in yellowness compared with that of the control group, which visually resulted in a more intense orange hue and suggested that the transfer of SP pigments from the diet to the meat was very efficient. In our study, the progressive increase in inclusion of SP in the diets resulted in a corresponding progressively more intense alteration in the color of the feeds (Table 2) and feces (Table 3), but this did not cause, as already described above, significant modifications of the meat color, probably because the color profile of pig meat, which is markedly different from that of broiler meat, is more capable of “masking” the effect of pigments possibly transferred from SP to meat.

Finally, the progressive replacement of SBM with SP did not affect the cumulative allocation of the intramuscular fat composition of loin meat into saturated, monounsaturated and polyunsaturated fatty acids in our study, and their contents were, in contrast, fully comparable among control-fed pigs and pigs fed different levels of SP. These results agree with the findings of Martins et al. [20], who reported that the inclusion of 10% SP in the diet did not modify the dietary fat categories of loins originating from 28 kg piglets. Conversely, in pigs slaughtered at 110 kg BW, Altmann et al. [21] reported similar saturated content but lower monounsaturated and greater polyunsaturated fatty acids in loin meat from pigs fed SBM-based diets or diets where SP replaced 50% to 100% SBM with SP. The fatty acid composition of meat is important for consumers’ health status, as excessive intake of saturated fatty acids has been regarded as a risk factor for heart disease [51], and data available for pig meat when SP is used as a feedstuff are still very limited. In this context, studies on broiler chickens have shown that the consumption of diets containing high levels of SP can impact the dietary fat categories of meat. Indeed, both Costa et al. [52] and Fernandes et al. [49] reported an increase in saturated fatty acids and a decrease in polyunsaturated fatty acids in the breast meat of broiler chicks fed diets including 15% SP compared with those fed conventional SBM-based diets. On the other hand, Altmann et al. [53] reported only minor differences in the fatty acid composition of thigh meat from chickens fed control diets or diets where 50% to 75% SBM was replaced by SP. Therefore, information on the effects of using SP as a feed ingredient in monogastric diets on the fatty acid composition of meat is still controversial and needs to be strengthened, also considering other relevant deposition tissues in animals, such as backfat and ham fat depots in the case of pigs. For this purpose, detailed fatty acid composition of fat from different tissues collected on these experimental pigs will be the topic of a further study of this project.

## Conclusions

The incorporation of SP in the diets to progressively replace SBM did not impair the feed intake, growth rate and feed efficiency of pigs, irrespective of the extent of such replacement. Similarly, carcass traits, the yield of dressed ham and other major commercial cuts were comparable across the different experimental dietary treatments and to those of pigs fed the soybean-based diet. The same was found for the chemical and physical attributes of loin meat.

In conclusion, the results obtained at the herd and slaughter levels suggest that SBM can also be completely replaced with SP powder in the feeds of growing and finishing pigs without leading to any adverse performance effects, thus providing new insights into the potential for switching growing pig feeding systems toward more environmentally sustainable diets. At the same time, further research is warranted to assess the effects of high levels of SP inclusion on pigs’ metabolic status and organoleptic attributes of meat obtained. Moreover, these dietary treatments should be tested also on other genetic types, that could be characterized by growth potential and nutritional needs different from those of hybrids used in the current study.

However, before this novel feed can be integrated into current production systems, the economic feasibility of such a replacement needs to be considered. Currently, the supply of SP is yet limited, and the price gap between SBM and SP is still large in favor of SBM. These issues will likely constitute in the near future the main critical area of improvement for the real implementation of SP in the diets of growing pigs, and future prospects should address to obtain a significant cost reduction and increased production efficiency in order to make SP competitive with other more established feeds.

## Data Availability

The datasets used and/or analyzed during the current study are available from the corresponding author on reasonable request.

## Abbreviations

ADG: Average daily gain
AMSA: American Meat Science Association
BF: Backfat depth
BW: Body weight
CIE: Commission Internationale de l’Eclairage
CP: Crude protein
DM: Dry matter
EE: Ether extract
FA: Fatty acid
FEFAC: The European Feed Manufacturers’ Federation
G:F: Gain to feed ratio
GC-FID: Gas Chromatography Flame Ionization Detector
LL: *Longissimus* *lumborum*
NRC: National Research Council
OPBA: Organismo Preposto al Benessere degli Animali
SBM: Soybean meal
SP: Spirulina
